# Becoming Food Aware in Hospital: A Narrative Review to Advance the Culture of Nutrition Care in Hospitals

**DOI:** 10.3390/healthcare3020393

**Published:** 2015-06-01

**Authors:** Celia Laur, James McCullough, Bridget Davidson, Heather Keller

**Affiliations:** 1 Applied Health Sciences, University of Waterloo, Waterloo, ON N2L 3G1, Canada; E-Mails: cvlaur@uwaterloo.ca (C.L.); jrmccull@uwaterloo.ca (J.M.); 2 Canadian Nutrition Society, Ottawa, ON K1C 6A8, Canada; E-Mail: bdavidson@cns-scn.ca; 3 Schlegel-University of Waterloo Research Institute for Aging, Waterloo, ON N2J 0E2, Canada

**Keywords:** malnutrition, best practice, hospital, older adults, education, training

## Abstract

The Nutrition Care in Canadian Hospitals (2010–2013) study identified the prevalence of malnutrition on admission to medical and surgical wards as 45%. Nutrition practices in the eighteen hospitals, including diagnosis, treatment and monitoring of malnourished patients, were *ad hoc*. This lack of a systematic approach has demonstrated the need for the development of improved processes and knowledge translation of practices aimed to advance the culture of nutrition care in hospitals. A narrative review was conducted to identify literature that focused on improved care processes and strategies to promote the nutrition care culture. The key finding was that a multi-level approach is needed to address this complex issue. The organization, staff, patients and their families need to be part of the solution to hospital malnutrition. A variety of strategies to promote the change in nutrition culture have been proposed in the literature, and these are summarized as examples for others to consider. Examples of strategies at the organizational level include developing policies to support change, use of a screening tool, protecting mealtimes, investing in food and additional personnel (healthcare aides, practical nurses and/or diet technicians) to assist patients at mealtimes. Training for hospital staff raises awareness of the issue, but also helps them to identify their role and how it can be modified to improve nutrition care. Patients and families need to be aware of the importance of food to their recovery and how they can advocate for their needs while in hospital, as well as post-hospitalization. It is anticipated that a multi-level approach that promotes being “food aware” for all involved will help hospitals to achieve patient-centred care with respect to nutrition.

## 1. Introduction

Forty-five percent of patients admitted to a medical or surgical ward in Canadian hospitals are malnourished, yet many remain undetected according to the Nutrition Care in Canadian Hospitals (NCCH) study (2010–2013) [1]. Malnutrition has been shown to independently increase mortality, length of stay and risk of readmission, affecting patient flow and, ultimately, healthcare costs [1,2]. In Canada, it was found that a malnourished patient’s cost of care was approximately $2000 more than a well-nourished patient’s care [3]. Undetected malnutrition is of particular concern both during admission and upon discharge, as patients who do not receive adequate nutrition care are at increased risk for complications [1].

The prevalence of hospital malnutrition is also high in other countries, with around 20%–50% of patients in acute care being malnourished, depending on the population and criteria for determination [4]. In the U.K., Nutrition Screening Weeks reported approximately one in three patients were at medium to high risk of malnutrition upon admission [2]. The Australasian Nutrition Care Day Survey (ANCDS) found that 32% of patients were malnourished, with these patients having a longer length of stay and higher readmission rates [5]. ANCDS also reported better referral for malnourished patients (>50% of wards surveyed); however, individual patient data were not reported [6]. Other work in this field suggests that referral processes are *ad hoc* [7], often missing malnourished patients. These studies demonstrate that nutrition care practices can vary and are inconsistent regarding screening, referral for diagnosis and treatment of patients who are malnourished.

Malnutrition has long been recognized as a serious clinical issue. Even as early as 1974, Butterworth indicated a high prevalence and advocated for the need for increased prevention efforts and focus on nutrition in medical education [8]. McWhirter *et al.* similarly reported high prevalence values in 1994 [9]. Despite a focus on hospital malnutrition over the past few decades, recent studies indicate that prevalence at admission remains high, and patients and hospitals continue to suffer consequences [1,4,6,9,10,11]. This suggests that strategies to change nutrition practice in and out of the hospital to date have not necessarily been effective.

Research has demonstrated the benefits of improved nutrition on health outcomes for malnourished patients [12]. For example, the NCCH study identified that malnourished patients on admission who improved their nutritional status had a shorter length of stay than those who did not improve [1]. Based on these consistently high prevalence rates, inconsistent care processes and the potential for these processes to improve outcomes, it is clear that change in nutrition practice is needed in hospital. Clearly, this is a problem that requires a comprehensive approach to promote a culture that is supportive of improved nutrition care practices through shared responsibility of hospital staff [1,7]. In Canada and internationally, an increase in awareness of the organization, staff, patients and their families around the importance of adequate food intake, barriers and enablers to food intake, as well as specific roles, will work towards improving nutrition culture [13]. In 2013, the Alliance to Advance Patient Nutrition published a call to action for improving nutrition care in hospitals, which lists several principles for action including the need to “create an institutional culture where all stakeholders value nutrition” [13].

The aim of this manuscript is to summarize existing literature and to identify processes and strategies to promote a “food-aware” culture in hospitals. Examples of strategies used by hospitals are anticipated to help other hospitals (organizations) move forward with their own culture change efforts.

## 2. Methods: Problem Identification and Scope of Narrative Review

Recent evidence supporting the need to change culture in Canadian hospitals is based on the prospective cohort study, Nutrition Care in Canadian Hospitals (NCCH), conducted by the Canadian Malnutrition Task Force (CMTF). The overall aim of NCCH was to determine the prevalence and potential causes of malnutrition in eighteen Canadian sites recruited to represent different provinces, communities and hospital type (academic *vs.* community) [1,7,14]. Data were collected to provide insight into the current nutrition culture in hospitals and direction for improvement. Results indicated that nutrition practices in these hospitals, including diagnosis, treatment and monitoring of malnourished patients, were *ad hoc*, and older patients were more likely to be malnourished and at increased risk of complications [1,7,15,16].

The NCCH also examined patient and site characteristics to determine the most common barriers to food intake, which included effects of illness (e.g., poor appetite), eating difficulties and organizational barriers (e.g., mealtime interruptions) [14,17]. A physician survey found that although the majority agreed that nutrition assessment should be performed at admission (87%), during hospitalization (86%) and at discharge (78%), most reported that this was rarely done in practice (admission 33%, during hospitalization 41%, at discharge 29%) [14,15]. When nurses were surveyed, they underestimated the prevalence of malnutrition [14,16]. Focus groups conducted with nutrition staff found the overarching theme that emerged was the need to develop a nutrition culture in hospitals; dietitians alone cannot change the attitudes and knowledge of other healthcare professionals needed to change culture [7]. These findings suggest that changes in nutrition practice must be supported by institutional culture, from an organization level, such as policies and support systems, to the individual level, such as attitudes and awareness of staff, including education regarding their roles. This culture could be developed by using valid tools, creating effective systems to support delivery of care, being responsive to care needs and uniting the right person with the right task [7]. Dietitians are a key component within this culture change; however, effective change will only be achieved through a multi-level approach that incorporates all hospital staff, patients and families.

Based on these findings, the CMTF decided to undertake an evidence-informed and consensus-based process to develop a feasible nutrition care algorithm that could be advocated as an improved practice in Canadian hospitals. The Integrated Nutrition Pathway for Acute Care (INPAC) resulted from a modified Delphi consensus process [18]. As an initial step in this modified Delphi, a focused narrative review was conducted to highlight: (1) the problem of nutrition care in hospitals; (2) nutrition care algorithms created to date; and (3) processes and strategies used to support a “food-aware” culture in hospitals. As other algorithms or guidelines were available as a starting point, with some based on graded systematic reviews of the literature [19,20], initial evidence synthesizing efforts were only undertaken to extend and update this literature. The articles in this narrative review were initially used to provide a common knowledge base for the Delphi participants. However, as these citations also recommended care processes and changes in practices, as well as examples for change, these aspects were further summarized in this review.

Two investigators (HK, BD) conducted a focused narrative review using numerous sources, including peer-reviewed literature, as well as websites of leading organizations with a mandate consistent with ameliorating malnutrition in the acute care setting (e.g., American Society of Parenteral and Enteral Nutrition, Academy of Nutrition and Dietetics, European Society of Parenteral and Enteral Nutrition, National Institute for Health and Care Excellence, British Association for Parenteral and Enteral Nutrition, Fight Malnutrition Now, *etc.*). Search terms included: “nutrition care”, “acute care”, “malnutrition”, “screening”, “assessment”, “treatment plans” and their derivations. The search was focused on citations after 2010, and Google Scholar and PubMed were the primary search engines. This date was chosen because a systematic review by the American Society of Parenteral and Enteral Nutrition was published in 2011 [19]. Key journal (e.g., Clinical Nutrition, Journal of Human Nutrition and Dietetics *etc.*) archives from 2010 onwards were also manually searched for pertinent titles. Only English language articles were used, resulting in a total of 57 citations considered. Any article that discussed strategies or processes to improve nutrition care was included, regardless of the quality of the citation or evidence demonstrating the efficacy of the strategy. This approach was taken as there are very few research studies demonstrating the efficacy of improved nutrition care processes. By summarizing these examples and the suggested ingredients for success in changing the nutrition culture, key factors considered essential can be elucidated for planning future culture change. All articles were reviewed with details on processes and strategies extracted and summarized. These points were further reviewed and synthesized by all authors, highlighting key articles, strategies and processes as examples for becoming “food aware”. 

## 3. Findings and Discussion

### 3.1. Confirmation of the Need for a Culture Change

The literature reviewed confirmed a number of organizational and environmental factors that contribute to malnutrition in hospitals. For example, lack of communication between patients, staff and food services led to patients receiving food they would not eat [17]. Furthermore, an ethnographic study of four acute hospital trusts in England and Wales identified several key challenges, including how hospitals are not necessarily designed or managed to support the diverse needs of particular patients and that organizational factors can make it difficult to provide the best care (including nutritional care) [21]. These, and others issues, may be addressed with a change in hospital culture towards promoting and improving nutrition. Identification and raised awareness of this problem and its impact on patient outcomes and healthcare utilization is a first step for building readiness for a change in nutrition culture.

### 3.2. A Multi-Level Approach Is Needed

The citations reviewed commonly pointed to the need to consider the problem of malnutrition from a multi-level view point, not only to more fully understand why malnutrition remains under-detected and treated, but also why malnutrition can develop in hospital [22,23,24,25,26,27]. A multi-level approach means that the problem arises from several levels of influence, including the patient, staff, unit, hospital and region. Consequently, to promote solutions to hospital malnutrition, a multi-level approach to changing care practices is needed [22,23,24,25,26,27].

Older adults are a subgroup of the hospital population that presents as more likely to be malnourished and to have significant barriers to food intake [17]. As a vulnerable group, there have been several initiatives focused on older patients described in the literature, which can help to elucidate ingredients for successful culture change. For example, a five-domain framework to promote senior-friendly hospitals has been suggested to address care issues, including: (1) organizational support; (2) processes of care considering older adult needs; (3) emotional and behavioural environment ensuring that older adults are valued and supported; (4) ethics in clinical care and research; and (5) physical environment supporting and enabling older adults [28]. These factors, which may be generalizable to other areas, such as nutrition care, support the contention that there is no single solution and that a multi-level approach is needed.

Two examples of multi-level approaches are the Seven Steps to End Malnutrition (U.K. Age Concern), and the Eight-Step Interdisciplinary Framework on the Prevention of Undernutrition (Victorian Government in Australia) [22,23,24]. These frameworks do not articulate details on how to implement these changes nor how policies and best practices can be realized; however, they do emphasize the need to address the issue from several levels, incorporating organization, staff and patients in the change process. Prior research notes that nutritional care requires inter-disciplinary teams [11,24,29,30], and it is the responsibility of all staff to be food aware [22,26,31,32]. All hospital staff, patients and their families must work together to change the culture of nutrition to improve the quality of care. Box 1 summarizes some components of multi-level strategies and processes to become food aware in hospital and to enhance the culture of nutrition.

### 3.3. Multi-Level Culture Change Is Feasible 

Whilst staff seem to recognize that malnutrition is an important problem in hospitals, there is a lack of a coordinated approach to nutrition care, including poor interdisciplinary communication and lack of a sense of shared roles and responsibility [33]. A study by Bell *et al.* in Australia is an example of how multi-disciplinary, multi-modal and multi-level strategies can be used to improve nutrition care and be systematically embedded into routine clinical practice [31]. The key components of this approach included: promoting nutrition as a medicine; a coordinated multi-disciplinary approach to meeting patients’ nutrition needs through recognition of malnutrition; improved communications and accountability of roles; and enhanced foodservice systems, including food provision at the bedside between meals. In patients with acute hip fractures, this approach decreased reliance on one practitioner and allowed nutrition assistants and dietitians to appropriately prioritize tasks. This multi-disciplinary and multi-department effort can lead to a change in processes and has the potential to significantly reduce barriers to food intake [31]. Although only applied to hip fracture patients, it is anticipated this approach would also be effective in other patient groups [31].

Box 1 **Overview of multi-level strategies and processes to become “food aware”.**

**Suggested Practices to Change the Culture of Nutrition Care****Organizational Level**
Hospital management aware of the effect nutrition status has on length of stay, risk of readmission, and cost to the hospital, so nutrition is made a priorityUse of knowledge translation/implementation frameworks to develop and implement policies/protocols for enhanced nutrition careFrameworks in place to support changes in nutrition practices/cultureHospital benchmarking and progress tracking for nutrition related goalsEffective communication systems (*i.e*., between wards and food services, and between health care professionals)Focus on all aspects of the nutrition care process including screening, referral, assessment, intervention, monitoringInterventions to promote intake (*i.e.*, use of colour coded trays for patients requiring feeding assistance or protected mealtimes)Food services is able to respond quickly to diet changes and allow food access outside of meal times (*i.e.*, snack carts)
**Staff Level**
Clarification of staff roles and responsibilities in nutrition careStaff education and training on how to perform these roles (*i.e*., nutrition screening)Auditing and feedback of nutrition care practicesIndividual actions to promote nutrition (*i.e.*, avoiding interruptions at mealtimes, providing feeding assistance if needed)Ensuring nutrition is considered in transitions in care (*i.e*., handovers, discharge or transfer to other wards/areas)Training of hospital volunteers to assist with specific tasks, when appropriateReminders in place for staff to ensure training is carried over into practice and changes are sustained
**Patient-Family Level**
Encouraging patient and family participation in nutrition care (*i.e.*, intake monitoring, advocating for nutrition needs, making the dining area as pleasant as possible)Educating patients and families on the importance of nutrition during and post hospitalizationTraining families on meal set-up and assistance for patientsAllow social interaction (*i.e.*, opportunities for patients to eat while family is present)

### 3.4. Using Implementation Frameworks to Support Culture Change

The Bell *et al.* study [27] along with other reviewed implementation studies [31,34] highlight the need for prospective use of frameworks and models during implementation [34,35,36,37,38,39,40]. These frameworks need to be flexible to the needs of the hospital and can adapt based on changing policies, staff, *etc.* [35,36]. Frameworks and models can be instrumental in ensuring all factors are considered and opportunities exist to adapt and revise the change strategies. A key example is the Knowledge-to-Action process, which takes the tools produced through knowledge syntheses and applies them in an Action Cycle that incorporates the context, barriers, monitoring and evaluation processes, eventually leading to sustained change [35,39]. This framework can also encompass a series of Plan-Do-Study-Act cycles to allow for testing and adapting of strategies of implementation [41]. Barriers to change need to be assessed, as well as potential solutions to overcome these barriers [35,39].

Understanding which implementation strategies have been effective and why is important for taking an inter-disciplinary approach to change nutrition culture, particularly at the organizational level. Detailed reporting of the implementation process and application of the framework is essential in the development of effective implementation strategies, which also allows for increased reproducibility [36,37]. As outlined in the PARIHS (Promoting Action on Research Implementation in Health Services) framework, evidence, context and facilitation are key to effective implementation [36,37]. The Michie COM-B (capability, opportunity and motivation) framework is another approach that has been used to facilitate change [42]. This model has behaviour change at the centre, surrounded by intervention functions and then by policy categories, indicating the need to incorporate policy that will enable the change in nutrition care practice, thus leading to sustained behaviour change [42]. Whichever implementation framework is chosen, it must be able to meet the needs of the local context and allow for sustained change.

### 3.5. The Organizational Level

Hospital management needs to sponsor the actions taken to improve the nutrition culture. Awareness is a first step, as hospital management may or may not be aware of the prevalence and implications of malnutrition. Online databases, such as Health System Evidence, exist to allow for management and policy makers to have access to the latest systematic reviews; however, access does not guarantee awareness or action [40]. Integrated knowledge translation and implementation strategies that include management are also needed.

Management must translate evidence into policy and protocols that support and sustain context-specific changes that revolve around patient centred care [43]. Knowledge translation tools, such as SUPPORT, Tools for evidence-informed health Policymaking (STP), have been used to increase evidence-based decisions in policy and may also be applicable for hospital management [40]. An example of policy is the U.K. Essential Standards for Quality and Safety that contend that any service provider must protect patients from the risks of inadequate food and fluid intake by: (1) providing appropriate and nutrient-dense food, sufficient to meet the patient’s needs; (2) meet religious and cultural needs; and (3) support intake of food and fluid, *i.e.*, ensure eating assistance [44]. Currently, there are no Canadian policies specific to nutrition care processes.

Management has ultimate control of budgets, thus support from them is essential to any change in culture. NCCH focus groups indicated that hospital budgets affected flexibility in the variety of the menu, food availability outside meal times and ability to meet diverse needs of patients [7]. Without the investment in food, staff is unable to improve the nutrition culture, nor can they provide patient centered care with respect to nutrition [7]. As noted in NCCH, this lack of hospital investment can spiral into low morale for dietitians and diet technicians [12].

Benchmarking that tracks progress towards implementing nutrition screening or removing barriers to food intake are useful organizational strategies in culture change. Audits and feedback are recognized mechanisms for changing and sustaining staff behaviour [45]. The British Association of Parenteral and Enteral Nutrition (BAPEN) Nutrition Screening Weeks is an example of how hospitals can benchmark themselves against others sites or units and may stimulate changes across sites and within teams [2].

Effective systems, including communication, referral processes and food delivery, have been noted as key areas for improvement [12]. Well-organized, accurate and timely communication within and between departments and wards is essential for patient-centred care in nutrition. For example, red trays highlight that the patient receiving this tray needs mealtime assistance [46]. A variety of other communication strategies, such as white boards in patient rooms and flags within electronic medical charts, are also available to support effective communications.

Food service management needs to be responsive to the care needs of the patients and be key contributors to improving nutrition culture [7,40]. To be more patient centred, food services needs to be flexible to provide quick responses to diet order changes, to have meals available when the patient is ready to consume them and to allow food access outside of mealtimes [12]. Specific examples where food service has been improved include the addition of mealtime assistance from volunteers or other available staff [12], implementation of selective mid-meal services (snacks) [27], treating food as medicine [27] and monitoring of food/fluid intake and body weight [47].

Use of a valid and reliable screening tool, such as the Canadian Nutrition Screening Tool (CNST), in acute care hospitals raises awareness and, more importantly, identifies patients early, so that nutrition care can be provided as needed [48]. To be effective, a positive screening result needs to be followed with a malnutrition diagnosis and, where required, individualized assessment and treatment [49]. Nutrition care algorithms [19,20], including the new Integrated Nutrition Pathway for Acute Care [18], are examples of processes that link screening to subsequent actions that can ensure appropriate referrals to dietetic staff and improve nutritional status through standardized processes. Optimizing and monitoring food intake to ameliorate poor nutrition is a key component [19]. If a patient’s length of stay is prolonged, risk should be re-assessed to identify any iatrogenic malnutrition [48].

Barriers to food intake, such as interrupted mealtimes, were reported in NCCH, as the most common challenge to food intake [17]. During mealtimes, staff should avoid interrupting patients, so they can have sufficient time and support to eat their meals. One strategy developed to address this barrier is protected mealtimes, which includes realigning nursing tasks around meals and eliminating nonessential interruptions [50]. Improved food intake is noted with additional staff, as well as just reorganizing mealtime tasks to promote food intake [50]. Hospitals need to examine what processes are needed to improve the situation in their own setting and ensuring that roles and responsibilities of pertinent staff include focusing on supporting food intake. Other studies have demonstrated mixed or even negative results for protected mealtimes, indicating that this policy may not be effective in all hospitals [51]. However, this change requires careful implementation and evaluation to ensure fidelity with the strategy [40]. As with screening, a policy is not sufficient to enact change, thus a multi-level approach is needed.

### 3.6. The Staff Level

Improving nutrition culture requires identifying the tasks best done by specific disciplines or personnel and having them accountable for these roles [7,37]. Specifically, nursing time is in high demand in hospital, as this front-line staff is key to every patient care process. Porters or food service personnel who deliver trays can have their role expanded to make sure the tray is within reach and packages are opened if so desired by the patient. This change leaves nursing available for roles that fit their expertise. For example, nutrition screening is a key task that is consistent with nursing scope of practice [13,25]. Role delineation is largely context specific, depending on the personnel available. Where feasible, having a key position focused on mealtime care can improve processes. For example, a paid nutrition assistant who can provide mealtime assistance has been found to improve food intake of patients [52]. This position could also troubleshoot a variety of strategies to support food intake, communicate with the dietitian and kitchen around individualized needs, manage ward stock, monitor food intake, assist with eating and encourage patients to eat [25].

Every staff member has a potential role to play. For example, cleaning staff can avoid cleaning patient rooms during meals, and a variety of staff can make the eating area as pleasant as possible (e.g., removing nonessential medical/care equipment from sight) [24]. Potential roles of staff may include: physicians assessing overall nutritional status, chart on status, monitoring the patient, involving the dietitian and considering nutrition at discharge planning; nurses can initiate nutrition care processes by screening, monitoring food intake and checking if eating assistance is needed; dietitians can do anthropometric measurements, assess the patient, start a care plan, re-evaluate and monitor the patient’s nutritional status; nutrition assistants can assist the dietitian as needed, assist with feeding and arrange meals; the occupational therapist can assess for eating challenges and provide adaptive equipment; the speech pathologist can assess for dysphagia and identify strategies to support safe swallowing; the physiotherapist can prescribe exercises to develop tolerance and strength for extended sitting and prescribe a safe seating position at meals; and the family caregiver can monitor and support food intake, providing assistance as needed [23,24]. There is also potential for hospital volunteers to be trained with specific tasks, when appropriate [22].

To carry out the specified roles, all staff and volunteers require training and time to learn and implement these roles. Training needs to be sufficient to promote sustainability and should be embedded into the orientation of new personnel. Evidence suggests that there is a mismatch between staff attitude and what is done in practice [53]. Staff needs to understand that they are accountable for the quality at which these roles are carried out [7], and auditing and feedback on practices can support uptake of roles [45]. Organizational policy and protocols can make it possible and desired for staff to complete designated care activities. Additionally, training should not be seen as a discrete event; reminders are suggested to reinforce training and assist in minimizing the gap between training and practice [40].

Training should adapt to the local context each time it is delivered. Each new edition can incorporate staff suggestions, making it more likely to be applied and effective in practice. Involving key opinion leaders in both the training and translation to practice is needed for implementation, as is having supportive hospital management for organizational changes [40]. Flexibility in implementation is also imperative, as is allowing for trial and error to find the most effective approach, through using frameworks, such as the Model for Improvement, and adapting accordingly [38,39,40]. It is also necessary to have nutrition education incorporated into the initial training of all healthcare professionals. This can include training in medical nutrition education, as well as in other professions, as the students of today can be the “nutrition champions” of tomorrow [54,55,56,57,58].

### 3.7. The Patient-Family Level 

Patients need to recognise that eating can be their way of contributing to their own recovery and that adequate food intake has a positive effect on their treatment goals and successful discharge. In 2013, an ethnographic study conducted in the U.K. found that the hospital environment resulted in feelings of dependence and low self-esteem in older adults [21]. Empowering patients through knowledge and understanding of how their care can be improved by treating food like medicine [32] is a possible strategy that may increase their autonomy.

In hospital, patients may become frustrated by an inability to eat independently and may be reluctant to ask for help from busy staff [21]. Staff needs to be aware of the barriers to food intake and understand mechanisms to avoid exacerbating the issue, as well as increase focus on patient-centred care. For example, staff can suggest to family members that they visit during mealtimes, which can ensure patients have assistance eating and social interaction while improving food intake without taking staff time [23,24]. Staff, volunteers or written material can be used to inform patients that they can and should ask for food (when appropriate) [59]. This combination of increased patient knowledge and awareness, as well as the ability of the hospital to provide adequate support, has the potential to improve patient experience and decrease complications associated with malnutrition. 

The importance of adequate nutrition does not end when a person is discharged from hospital, and this must be clear to the patient, family and the discharge team [23]. Having the hospital provide nutrition-related materials at discharge specific to the patient’s needs may assist with this transition. This can include information regarding support services available in the community (e.g., home delivery meal programs) or information for their general practitioner, which will assist in their recovery. This is in line with the call to action that recommends the development of a comprehensive discharge nutrition care and education plan, as patients are rarely educated about their nutritional status before discharge [13]. Increasing awareness and providing education is only effective if the support mechanisms are in place to allow for adequate food intake.

## 4. Conclusions

Several initiatives and research studies identified in this narrative review have indicated the need for improvements in nutrition culture. A multi-level approach is a consistent recommendation for becoming food aware [1,7,13]. Examples of strategies and processes at the organizational, staff and patient levels have been provided to demonstrate that a change in culture to improve patient centred nutrition care is within reach. A structured implementation program using implementation frameworks is needed to change organizational policies and procedures, provide staff role delineation and training, as well as strategies to reinforce this training and to empower patients and families. Future implementation studies based on these strategies and processes summarized from the literature are needed to demonstrate not only the process of implementation, but also the effectiveness of the multi-level approach to becoming food aware. 
